# Epidemiological Interactions between Urogenital and Intestinal Human Schistosomiasis in the Context of Praziquantel Treatment across Three West African Countries

**DOI:** 10.1371/journal.pntd.0004019

**Published:** 2015-10-15

**Authors:** Sarah C. L. Knowles, Bonnie L. Webster, Amadou Garba, Moussa Sacko, Oumar T. Diaw, Alan Fenwick, David Rollinson, Joanne P. Webster

**Affiliations:** 1 Department of Infectious Disease Epidemiology, Imperial College London, St. Mary’s Campus, London, United Kingdom; 2 Department of Life Sciences, Imperial College London, Silwood Park Campus, Ascot, Berkshire, United Kingdom; 3 Natural History Museum, Parasites and Vectors Division, Department of Life Sciences, London, United Kingdom; 4 Réseau International Schistosomose, Environnement, Aménagement et Lutte (RISEAL), Niamey, Niger; 5 Institut National de Recherche en Santé Publique (INRSP), Ministère de la Santé, Bamako, Mali; 6 Institut Sénégalais de Recherches Agricoles (ISRA), Bel Air, Dakar, Sénégal; 7 Department of Pathology and Pathogen Biology, Centre for Emerging, Endemic and Exotic Diseases (CEEED), Royal Veterinary College, University of London, London, United Kingdom; Swiss Tropical and Public Health Institute, SWITZERLAND

## Abstract

**Background:**

In many parts of sub-Saharan Africa, urogenital and intestinal schistosomiasis co-occur, and mixed species infections containing both *Schistosoma haematobium* and *S*. *mansoni* can be common. During co-infection, interactions between these two species are possible, yet the extent to which such interactions influence disease dynamics or the outcome of control efforts remains poorly understood.

**Methodology/Principal Findings:**

Here we analyse epidemiological data from three West African countries co-endemic for urogenital and intestinal schistosomiasis (Senegal, Niger and Mali) to test whether the impact of praziquantel (PZQ) treatment, subsequent levels of re-infection or long-term infection dynamics are altered by co-infection. In all countries, positive associations between the two species prevailed at baseline: infection by one species tended to predict infection intensity for the other, with the strength of association varying across sites. Encouragingly, we found little evidence that co-infection influenced PZQ efficacy: species-specific egg reduction rates (ERR) and cure rates (CR) did not differ significantly with co-infection, and variation in treatment success was largely geographical. In Senegal, despite positive associations at baseline, children with *S*. *mansoni* co-infection at the time of treatment were less intensely re-infected by *S*. *haematobium* than those with single infections, suggesting competition between the species may occur post-treatment. Furthermore, the proportion of schistosome infections attributable to *S*. *mansoni* increased over time in all three countries examined.

**Conclusions/Significance:**

These findings suggest that while co-infection between urinary and intestinal schistosomes may not directly affect PZQ treatment efficacy, competitive interspecific interactions may influence epidemiological patterns of re-infection post-treatment. While re-infection patterns differed most strongly according to geographic location, interspecific interactions also seem to play a role, and could cause the community composition in mixed species settings to shift as disease control efforts intensify, a situation with implications for future disease management in this multi-species system.

## Author Summary

In many parts of Africa both urinary and intestinal schistosomiasis are endemic, and mixed species infections can be common. However, little is known about potential within-host interactions between the causative parasites, *S*. *haematobium* and *S*. *mansoni*, and how these might influence treatment success and post-treatment patterns of re-infection. Here, we bring together datasets from three West African countries to examine the epidemiological evidence for interactions between these two schistosome species relevant to the impact of treatment programmes using praziquantel (PZQ). Encouragingly, PZQ efficacy (in a double 40mg/kg dose format) was not significantly altered by co-infection, though since co-infections tended to be heavier, complete clearance was less likely than for single species infections. Despite positive associations in infection intensity for these two species at baseline, Senegalese children that were successfully treated for *S*. *haematobium* showed less intense re-infection if they were co-infected with *S*. *mansoni* at the point of treatment. Furthermore, in all three settings, the proportion of infections attributable to *S*. *mansoni* increased over successive rounds of PZQ treatment. These data suggest asymmetric competition may occur between *S*. *haematobium* and *S*. *mansoni* in the context of drug treatment, which may alter schistosome species composition as PZQ-based control programmes proceed.

## Introduction

Globally, at least 230 million people are estimated to have schistosomiasis [1]. In sub-Saharan Africa where the disease burden is highest, *Schistosoma haematobium and S*. *mansoni*, causing urogenital and intestinal schistosomiasis respectively, frequently overlap in their geographic distribution [2–5] as do their respective snail hosts *Bulinus* and *Biompharia* spp. In such areas, mixed species infections can be common [6–10], and may be even more widespread than currently recognised if diagnostic methods with greater sensitivity than standard microscopy are applied [11].

Co-infection with both *S*. *haematobium* and *S*. *mansoni* generates the potential for within-host parasite interactions, whereby the presence of one species may alter the course of infection or disease caused by the other. Such interactions could arise through competition for nutrients or mates, or immune-mediated mechanisms, including cross-reactive immune responses. Immune-mediated interactions may also arise through or be affected by drug treatment, if species differ in drug susceptibility, or if the drug in question alters host immunity in a way that favours one species over another [12, 13]. Although rarely investigated, such interspecific interactions may have important implications for schistosomiasis epidemiology, associated morbidity, and the effectiveness of control measures including PZQ treatment, the cornerstone of current schistosomiasis control programmes [14].

Evidence for biologically relevant interactions between co-infecting *S*. *haematobium* and *S*. *mansoni* comes from studies in animal models as well as humans. These two species can engage in mate competition, since during co-infection, infertile interspecific mating pairs form resulting in the release of ectopic eggs: *S*. *haematobium* eggs in faeces or *S*. *mansoni* eggs in urine [7, 15–17]. Moreover, mixed species infections produce different morbidity profiles in humans, altering the relative levels of bladder and liver morbidity [6, 8, 15, 18, 19]. Immune-mediated competition between *S*. *haematobium* and *S*. *mansoni* has also been reported in animal models [20–22] and immunological studies suggest widespread cross-reactivity among antigenic epitopes from different schistosome species [23].

However, exactly how such interactions might play out in epidemiological settings, and in the context of mass PZQ treatment, remains underexplored. In parts of Africa the species composition of schistosomiasis infections has shifted notably over time. At sites in Senegal, Niger, Cameroon and Egypt, *S*. *mansoni* has been introduced through changes in irrigation (e.g. dam construction), and has been seen to increase in prevalence and subsequently ‘take over’ from *S*. *haematobium* 24–29]. While changes in the distribution and relative abundance of *Biomphalaria* and *Bulinus* snails following water resource development have undoubtedly played a key role in such shifts [25, 28, 30, 31], whether and how within-host interactions between *S*. *haematobium* and *S*. *mansoni* might influence schistosomiasis epidemiology remains to be fully investigated. PZQ treatment could also alter the relative abundance of these two species, if drug efficacy varies between species or during co-infection, or if treatment alters interspecific interactions during re-infection [14]. With increasing momentum behind scaling up schistosomiasis control programmes across much of Africa [32], there is a clear need to understand how each species responds to treatment, whether these responses depend on the parasite community context, and the implications for epidemiology and morbidity.

Here, we use three epidemiological datasets from co-endemic areas of West Africa (Senegal, Niger and Mali) to investigate potential interactions between *S*. *haematobium* and *S*. *mansoni* in the context of PZQ treatment. In particular, we examine whether PZQ efficacy is altered by co-infection, the impact of co-infection on individual re-infection post-treatment, and how schistosome species composition, as well as prevalence and mean intensity of infection changes over the course of successive treatment rounds at the population level.

## Methods

### Study sites and datasets

**Fig 1:**
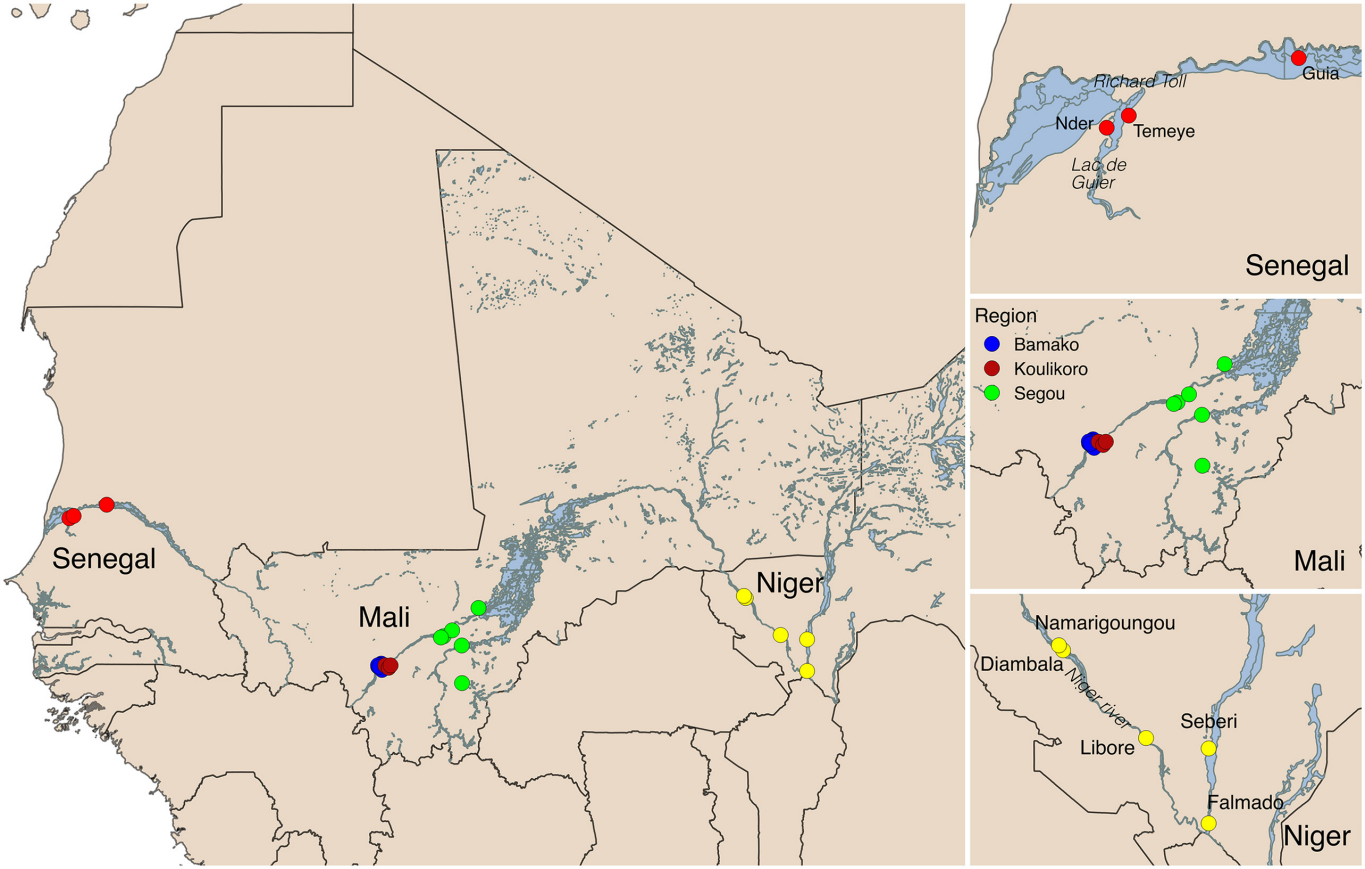
Map of the study sites in Senegal, Niger and Mali.

Two of the datasets analysed here come from co-endemic villages in Niger [4] and Senegal [5] collected as part of the CONTRAST project, while the third comes from sentinel sites monitored as part of Mali’s schistosomiasis control programme monitoring and evaluation activities [6]. These datasets are described in more detail below, their characteristics are summarised in Table 1, and their locations are shown in Fig 1.

**Table 1:** Characteristics of study sites in Senegal, Niger and Mali used in this study.

Country	Study type	Site/s	N children	Baseline uninfected (%)	Baseline single *S*. *haematobium* (%)	Baseline single *S*. *mansoni* (%)	Baseline co-infected (%)
Senegal	PZQ efficacy	Temeye	89	0	21.3	19.1	59.6
		Nder	107	0	0	2.8	97.2
Niger	PZQ efficacy	Diambala	180	0	22.2	24.4	53.3
		Namarigoungou	223	0	13.5	40.4	46.2
Mali	Monitoring & evaluation of national treatment programme	29 co-endemic schools in three regions (Bamako, Koulikoro and Ségou), 20 of which followed annually for 3 years.	2477	28.5	45	5.5	21

#### Senegal

Data were collected in 2007–8 from two villages (Nder and Temeye) in the Senegal River Basin (Fig 1). In previous work [5], village-level variation in PZQ efficacy and re-infection dynamics was documented over a one-year period at these sites. Here, we extend analysis of these data to consider the influence of individual co-infection status on PZQ efficacy, parasite clearance and re-infection dynamics. Full details on these sites and study design are given in [5], but a brief description of the study design follows. At baseline, children aged 5 to 15 were recruited in each village, given a unique identification number, and asked to provide a single urine and stool sample on three consecutive mornings. *S*. *haematobium* egg counts were made from filtrations of 10 ml of urine using the standard urine filtration method, and duplicate Kato-Katz thick smears were examined from each stool sample in order to calculate the number of *S*. *mansoni* eggs per gram of stool. Only children infected with either *S*. *haematobium*, *S*. *mansoni*, or both species were recruited into the follow-up study if consenting, and all infected children were then treated with two 40mg/kg doses of PZQ, spaced 3 weeks apart. Follow-up surveys were conducted at 6 weeks after baseline/the first PZQ dose (to monitor PZQ efficacy), 6 months from baseline and 12 months from baseline. All children were screened for both *S*. *haematobium* and *S*. *mansoni* at each time point, using the same diagnostics used at baseline. No treatment was given at the 6 week follow-up, but all children were given two 40mg/kg PZQ doses 3 weeks apart at both the 6 and 12 month follow ups. For *S*. *haematobium*, the number of eggs per 10ml urine was calculated for each sample, and infection intensity taken as the mean of these values across all available samples from a 3-day sampling period. For *S*. *mansoni*, infection intensity was taken as the mean number of eggs per gram of stool across all samples from the 3-day sampling period.

#### Niger

Data were collected in 2007–8 from two *S*. *haematobium*/*S*. *mansoni* co-endemic villages situated in the western part of the country along the Niger River—Diambala and Namarigoungou. The study design was very similar to the Senegal study described above, and is described fully in [4]. Briefly, children aged 6 to 15 infected with either *S*. *haematobium* or *S*. *mansoni* were recruited into the study at baseline, given unique identifiers, and follow-up surveys were conducted at 6 weeks, 6 months and 12 months from baseline. As in Senegal, two 40mg/kg doses of PZQ were given to all infected children after recruitment, and to all children in the study at the 12-month sampling point, irrespective of infection status. However, unlike in Senegal, no treatment was given 6 months from baseline. At each survey time-point, all children were screened for both *S*. *haematobium* and *S*. *mansoni* using the same diagnostic techniques used in the Senegal study, with the exception that one Kato-Katz slide was read per stool sample rather than two.

#### Mali

The Malian data analysed here formed part of the Monitoring and Evaluation component of the Mali National Schistosomiasis Control Program, supported by the Schistosomiasis Control Initiative (SCI). In 2004, a set of 33 schools (sentinel sites) were randomly selected from all schools in three regions known a priori to be highly endemic for schistosomiasis: Bamako, Ségou, and Koulikoro. Only the 29 schools that were co-endemic for *S*. *haematobium* and *S*. *mansoni* at baseline are included in the analyses presented here, as our focus is on individual level determinants of infection traits, rather than site to site variation in co-endemicity. Baseline data were collected in 2004. At each school, 50–110 children (approximately equal numbers of boys and girls) aged 7 to 14 years old were recruited, irrespective of infection status. Participants were asked to provide a single stool sample, and two urine samples on two consecutive days. Urine filtration and Kato-Katz examinations were carried out as described in [33], and infection intensity was calculated as the arithmetic mean number of *S*. *haematobium* eggs per 10ml urine, or mean number of *S*. *mansoni* eggs per gram of stool. Two subsequent follow-up surveys were conducted on this cohort in 2005 and 2006, immediately prior to annual PZQ administration by the national control programme.

### Statistical analyses

All statistical analyses were performed in R v3.1.1. Descriptive statistics (prevalence, mean infection intensity and confidence intervals) were calculated using the *survey* package [34], accounting for clustering of the data by school or village where necessary. The significance of model terms was assessed using likelihood ratio tests, which compared full models to models excluding the term of interest.

#### Baseline associations between *S*. *haematobium* and *S*. *mansoni*

First, we tested whether the likelihood of infection by each schistosome species depended on co-infection with the other, using baseline data from Mali where children were recruited irrespective of infection status. Binomial generalized linear mixed models (GLMMs) with a logit link were performed using the g*lmer* function in package *lme4*, with either *S*. *haematobium* or *S*. *mansoni* infection status as the response, and school fitted as a random intercept term. All children at the 29 co-endemic Malian schools surveyed at baseline were included. Co-infection status was coded according to WHO infection intensity categories: for *S*. *haematobium*, uninfected, light (<50 eggs/10ml urine) or heavy (≥50eggs/10ml urine) infection, and for *S*. *mansoni*, uninfected, light (1–99 epg), moderate (100–399 epg), or heavy (≥400 epg) infection. Linear and quadratic terms for age (mean-centred) were included as covariates, as well as gender and geographic region (as a 3-level factor). An interaction term between region and co-infection status was also examined to test for geographic heterogeneity in schistosome species associations. For all countries we also assessed whether baseline infection intensity was associated with co-infection, by modelling log-transformed egg counts for a given species as a function of co-infection status, age, gender and location (as a 2-level factor for village in both Senegal and Niger, and a 3-level factor for region in Mali). These infection intensity analyses included infected children only.

#### PZQ efficacy and parasitological cure

Data on the immediate effect of PZQ (6 weeks after the first of two 3-week spaced treatments) were available for four villages in Senegal and Niger. To examine whether co-infection might influence PZQ efficacy, for each country we calculated the egg reduction rate (ERR) for each schistosome species, in single and co-infected individuals respectively. ERR was calculated using the formula recommended in WHO guidelines [35], as the difference between the (arithmetic) mean pre- and post-intervention egg counts divided by the mean pre-intervention egg count, multiplied by 100. The calculation included only individuals infected with the focal species at baseline, with known baseline co-infection status, and for which an egg count at 6 weeks post-treatment was available. Although a wide range of formulas has been used in the past to calculate ERR, this formula was used as it has been shown to be the most robust metric for evaluating drug efficacy [36, 37]. Permutation tests were used to examine whether ERR differed significantly (p<0.05) according to co-infection status. We also calculated species-specific cure rates (CR)—the proportion of infected individuals that cleared infection six weeks after the first PZQ dose, by country and co-infection status. With the exception of *S*. *haematobium* in Niger (for which parasitological cure was almost universal) we modelled factors affecting individual parasite clearance 6 weeks after PZQ using binomial GLMs. Alongside co-infection status as the key predictor of interest, a number of covariates were controlled for in these models, including initial infection intensity of the focal species (as logged epg or eggs/10ml urine) to control for differences in efficacy arising from varying infection intensity and village.

#### Longitudinal dynamics and re-infection

Using the Mali dataset, we examined whether individuals’ probability of infection changed across annual rounds of PZQ treatment, according to their initial co-infection status. These analyses involved a subset of 20 schools from the original 29 co-endemic sentinel sites, as these had follow-up data for both *S*. *haematobium* and *S*. *mansoni* from all three years. We constructed a binomial GLMM, with *S*. *haematobium* or *S*. *mansoni* infection status as the response, and school and child ID included as random intercept terms. Fixed effects for baseline co-infection status (as a 3 or 4-level factor, depending on the species), year (as a 3-level factor: baseline, first and second follow-up) and their interaction term were used to examine whether temporal changes in infection for each species differed according to initial co-infection status. Region, gender and mean-centred age at baseline (linear and quadratic term) were fitted as covariates. Only children monitored at all three time-points (baseline, first and second follow-up years) were included, such that we analysed changes in infection probability over time in the same set of individuals. To examine whether drop-out from the Malian cohort might be biased according to children’s infection status at baseline, we used a binomial mixed model to test which baseline variables predicted whether children had full follow-up data. School was fitted as a random intercept term, alongside *S*. *haematobium* and *S*. *mansoni* infection categories, with region, gender and mean-centred age at baseline (linear and quadratic term) as covariates.

In Senegal and Niger it was also possible to separate effects of co-infection on parasite clearance from those on re-infection (the latter potentially including recommencement of egg-shedding by uncleared worms), since individuals were monitored six weeks after PZQ treatment. For each schistosome species, we used a binomial GLM with a logit link to test whether, among individuals infected at baseline but clear of infection 6 weeks later (after PZQ treatment), the probability of being re-infected 6 months from baseline depended on baseline co-infection status (single vs. co-infected). Covariates included were village, baseline intensity category for the focal species (2-level factor for *S*. *haematobium*: light vs. heavy; 3-level factor for *S*. *mansoni*: light, moderate or heavy), gender and mean-centred age (as a linear and quadratic term). An interaction term between village and co-infection status was also included to test for geographic heterogeneity in the influence of co-infection. Among the same individuals, we also used negative binomial GLMs (using the *glm*.*nb* function in the *MASS* package) to examine whether the intensity of re-infection at six months (eggs/gram stool for *S*. *mansoni*, or eggs/10ml urine for *S*. *haematobium*) was dependent on co-infection status at baseline, including the same covariates used in the binomial re-infection models.

### Ethics statement

Full details on ethical approval granted are provided in the original publications from which these data were gathered. In brief, ethical approval was obtained from the St Mary’s Hospital Local Ethics Research Committee, R&D office (part of the Imperial College, London Research Ethics Committee (ICREC; EC NO: 03.36. R&D No: 03/SB/033E) in combination with the ongoing CONTRAST and SCI activities. Within Niger, Senegal and Mali, all aspects of sample collections were carried out in the framework of the disease control activities implemented and approved by the Ministry of Health (MOH) and adopted by regional and local administrative and health authorities. In Senegal and Niger, the communities of the selected villages were informed about the objectives, the methodology of the study and the advantages. A meeting was organized with the population and verbal community consent was obtained for each selected village. Written and verbal consent was also obtained from school directors and teachers, as well as the children’s parents, prior to the recruitment of the children. Verbal assent was given by every child and their acceptance documented. Participation was voluntary and children could withdraw or be withdrawn from the study at any time without obligation. Results of the different diagnostic procedures performed on children were briefly explained to them. In Senegal and Niger, all children diagnosed as infected with schistosomiasis were immediately treated with 40mg/kg. In Mali, all children at participating schools were treated shortly after each survey, as part of the national schistosomiasis control programme activities.

## Results

### Baseline associations between *S*. *haematobium* and *S*. *mansoni*

**Fig 2:**
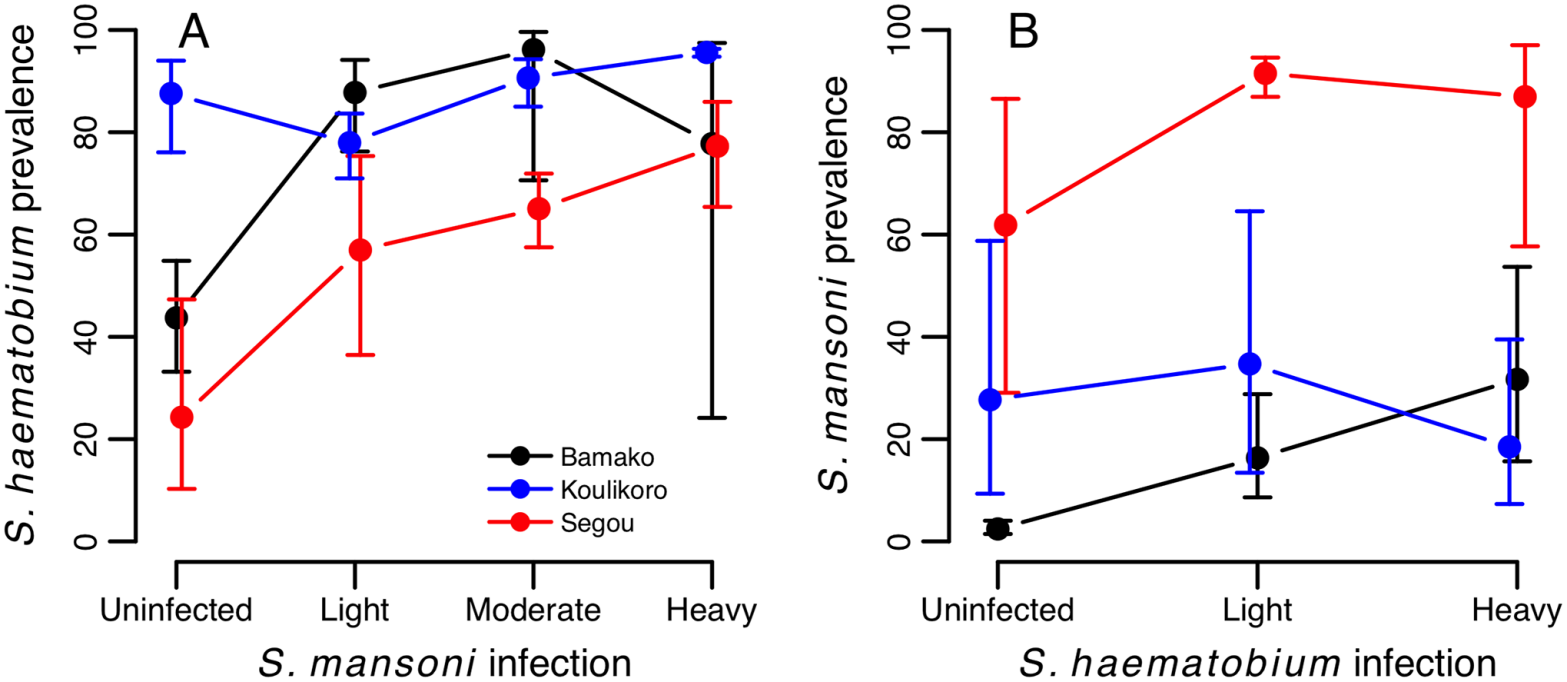
Baseline differences in infection probability for (A) *S*. *haematobium* and (B) *S*. *mansoni* among Malian children according to intensity of co-infection. Raw data are plotted with 95% exact confidence intervals, which account for clustering of prevalence data by school. Patterns are plotted separately for each of the three regions surveyed in Mali.

**Fig 3:**
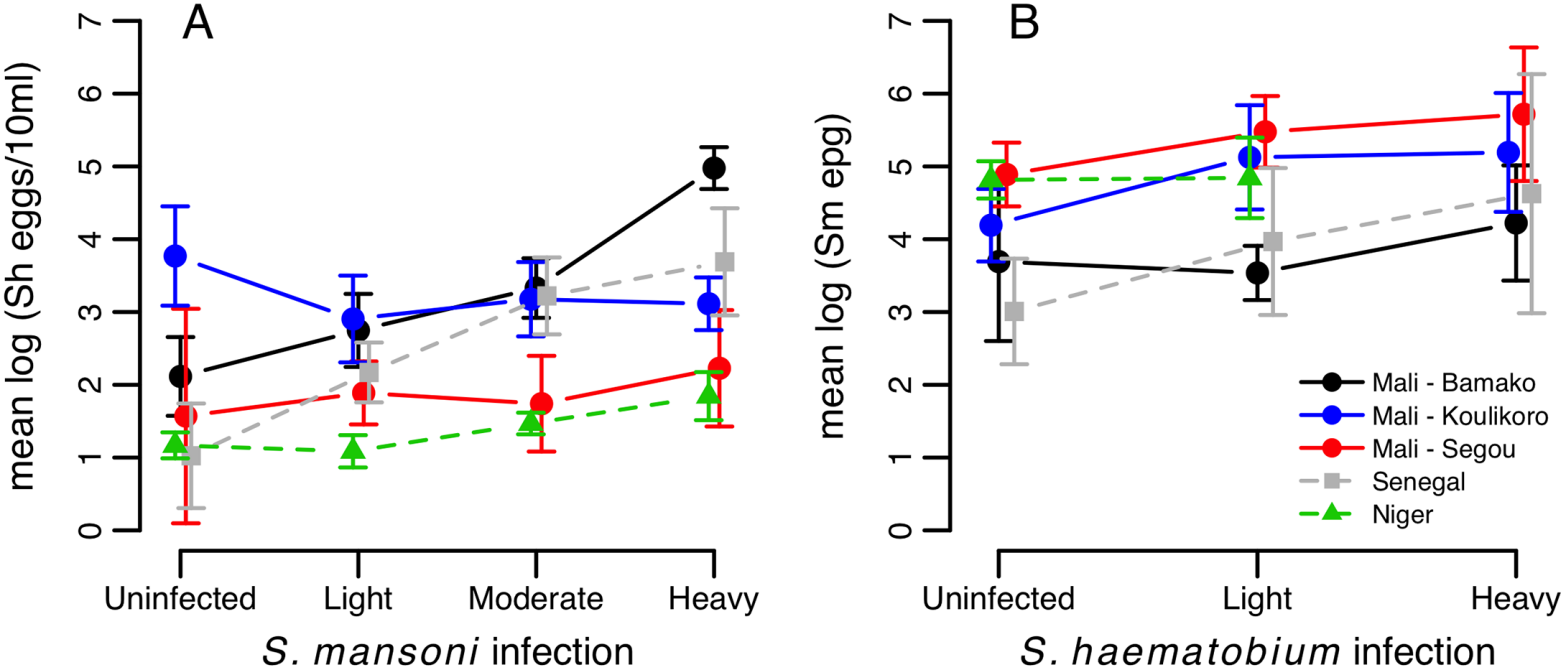
Infection intensity at baseline of (A) *S*. *haematobium* and (B) *S*. *mansoni* according to co-infection status in three West African countries. Arithmetic mean log-transformed egg counts are plotted for each species among infected children only, with 95% confidence intervals that account for clustering of the data by school in Mali, and by village in Senegal and Niger.

At baseline, associations between the two schistosome species were generally positive, although varied in strength across countries and regions. In Mali, infection probability for both schistosome species was positively predicted by the level of infection with the other species, after controlling for age and gender effects (Table 2). This relationship varied in strength across geographical regions (*S*. *haematobium*: *S*. mansoni*region interaction χ^2^_6_ = 22.28, p = 0.001; *S*. *mansoni*: *S*. haematobium*region interaction: χ^2^_4_ = 12.63, p = 0.013, Fig 2). Among infected individuals, infection intensity increased with the intensity of co-infection for both schistosome species, although again the strength of this relationship varied across countries and regions (Table 3; Fig 3). Across the 29 schools in Mali, these positive relationships for both *S*. *haematobium* and *S*. *mansoni* infection intensity were usually observed within each school, with few exceptions (Fig A in S1 Text).

**Table 2:** Baseline predictors of (A) *S*. *haematobium* and (B) *S*. *mansoni* infection probability across 29 co-endemic schools in Mali (n = 2477 children). Parameter estimates (on the logit scale) are from binomial mixed models. χ^2^ and p values are from likelihood ratio tests comparing models with and without the term in question. Age was mean-centred in both analyses. ‘ref’ indicates the reference level of each factor.

Variable	df	Parameter estimate (SE)		χ^2^	p
**(A) *S*. *haematobium***					
(Intercept)			0.016 (0.360)		
Age	1		0.105 (0.024)	18.760	<0.0001
Age^2^	1		-0.010 (0.012)	0.766	0.3816
Sex	1	Male (ref)	0	2.363	0.1243
		Female	-0.162 (0.105)		
*S*. *mansoni* infection	3	Uninfected (ref)	0	51.066	<0.0001
		Light	0.914 (0.207)		
		Moderate	1.411 (0.276)		
		Heavy	1.940 (0.306)		
Region	2	Bamako (ref)	0	19.015	<0.0001
		Koulikoro	-0.894 (0.759)		
		Ségou	2.345 (0.545)		
**(B) *S*. *mansoni***					
(Intercept)			-3.391 (0.562)		
Age	1		0.161 (0.035)	21.817	<0.0001
Age^2^	1		0.006 (0.017)	0.123	0.7253
Sex	1	Male (ref)	0	2.808	0.0938
		Female	-0.255 (0.149)		
*S*. *haematobium* infection	2	Uninfected (ref)	0	56.630	<0.0001
		Light	1.054 (0.202)		
		Heavy	1.861 (0.253)		
Region	2	Bamako (ref(A)	0	13.499	0.0012
		Koulikoro	4.441 (1.118)		
		Ségou	0.127 (0.807)		

**Table 3:** Baseline predictors of infection intensity for (A) *S*. *haematobium* and (B) *S*. *mansoni* across three co-endemic countries in West Africa. Results are from Gaussian GLMs (Senegal and Niger), or GLMMs with school as a random intercept term (Mali), using log transformed egg counts among infected individuals only (eggs/10ml urine or eggs/gram stool) as the response. χ^2^ and p values show results of likelihood ratio tests. Parameters from full models are shown, and age was mean-centred in analyses. ‘ref’ indicates the reference level of each factor.

	**Mali (n = 1398)**					**Senegal (n = 171)**					**Niger (n = 269)**				
**Variable**	**df**	**Parameter estimate (SE)**		**χ** ^**2**^	**p**	**df**	**Parameter estimate (SE)**		**χ** ^**2**^	**p**	**df**	**Parameter estimate (SE)**		**χ** ^**2**^	**p**
**(A) *S*. *haematobium intensity***															
(Intercept)			3.032 (0.224)					-0.179 (0.600)					1.244 (0.131)		
Age	1		-0.032 (0.019)	3.314	0.0687	1		0.014 (0.047)	0.172	0.6785			0.032 (0.028)	1.607	0.2049
Age^2^	1		-0.013 (0.009)	2.071	0.1502	1		0.005 (0.015)	0.129	0.7192	1		0.002 (0.013)	0.020	0.8890
Sex	1	Male (ref)	0	12.232	0.0005	1	Male (ref)	0	0.830	0.3623	1	Male (ref)	0	2.745	0.0976
		Female	-0.283 (0.081)				Female	0.257 (0.289)				Female	-0.169 (0.103)		
*S*. *mansoni* infection	3	Uninfected (ref)	0	15.967	0.0012	3	Uninfected (ref)	0	35.296	<0.0001	3	Uninfected (ref)	0	16.727	0.0008
		Light	0.399 (0.149)				Light	1.684 (0.500)				Light	-0.059 (0.142)		
		Moderate	0.591 (0.198)				Moderate	3.040 (0.588)				Moderate	0.276 (0.131)		
		Heavy	0.813 (0.202)				Heavy	3.513 (0.657)				Heavy	0.668 (0.200)		
Region / Village	2	Bamako (ref)	0	11.167	0.0038	1	Nder (ref)	0	8.633	0.0033	1	Diambala (ref)	0	0.070	0.7908
		Kouikoro	-0.632 (0.474)				Temeye	1.039 (0.357)				Namarigoungou	0.028 (0.108)		
		Ségou	0.830 (0.318)												
**Mali (n = 654)**						**Senegal (n = 172)**				**Niger (n = 333)**				
**df**	**Parameter estimate (SE)**		**χ** ^**2**^	**p**	**df**	**Parameter estimate (SE)**		**χ** ^**2**^	**p**	**df**	**Parameter estimate (SE)**		**χ** ^**2**^	**p**
**(B) *S*. *mansoni***															
(Intercept)			3.463 (0.322)					0.376 (11.233)					4.506 (0.131)		
Age	1		0.123 (0.024)	26.700	<0.0001	1		0.054 (0.032)	2.178	0.1400	1		0.119 (0.027)	21.450	<0.0001
Age^2^	1		0.002 (0.012)	0.017	0.8952	1		-0.011 (0.010)	1.227	0.2680	1		0.003 (0.013)	0.045	0.8323
Sex	1	Male (ref)	0	11.280	<0.0001	1	Male (ref)	0	1.957	0.1618	1	Male (ref)	0	0.687	0.4073
		Female	-0.361 (0.108)				Female	0.281 (0.205)				Female	-0.085 (0.104)		
*S*. *haematobium* infection	2	Uninfected (ref)	0	38.585	<0.0001	3	Uninfected (ref)	0	14.790	0.0006	3	Uninfected (ref)	0	1.664	0.4351
		Light	0.623 (0.143)				Light	0.189 (0.341)				Light	0.099 (0.106)		
		Heavy	1.106 (0.175)				Heavy	1.025 (0.362)				Heavy	-0.749 (0.948)		
Region / Village	2	Bamako (ref)	0	12.864	0.0016	1	Nder (ref)	0	36.943	<0.0001	1	Diambala (ref)	0	24.310	<0.0001
		Kouikoro	1.848 (0.470)				Temeye	-1.464 (0.233)				Namarigoungou	0.527 (0.106)		
		Ségou	0.667 (0.390)												

### Co-infection and the impact of PZQ

In both Senegal and Niger, egg reduction rates 6 weeks after the first PZQ dose did not differ significantly according to co-infection status, for either *S*. *haematobium* or *S*. *mansoni* (Table 4). Thus, there was no evidence that co-infection altered the efficacy of double dose PZQ.

**Table 4:** Egg reduction rate (ERR) and cure rate (CR) by species and individual co-infection status among children in Niger and Senegal. p-values comparing ERRs in single and co-infected subsets of individuals were derived using permutation tests. Numbers of individuals involved in each calculation are shown in brackets.

	Egg reduction rate (ERR)			Cure rate (CR)	
	Single	Co-infected	p (permutation test)	Single	Co-infected
***S*. *haematobium***					
Niger	98.6% (34)	99.4% (104)	0.515	94.1% (34)	98.1% (104)
Senegal	98.5% (18)	97.6% (146)	0.657	83.3% (18)	44.4% (146)
***S*. *mansoni***					
Niger	84.8% (79)	78.9% (104)	0.304	63.3% (79)	56.7% (104)
Senegal	99.3% (19)	98.5% (142)	0.576	57.9% (19)	44.4% (142)

**Fig 4:**
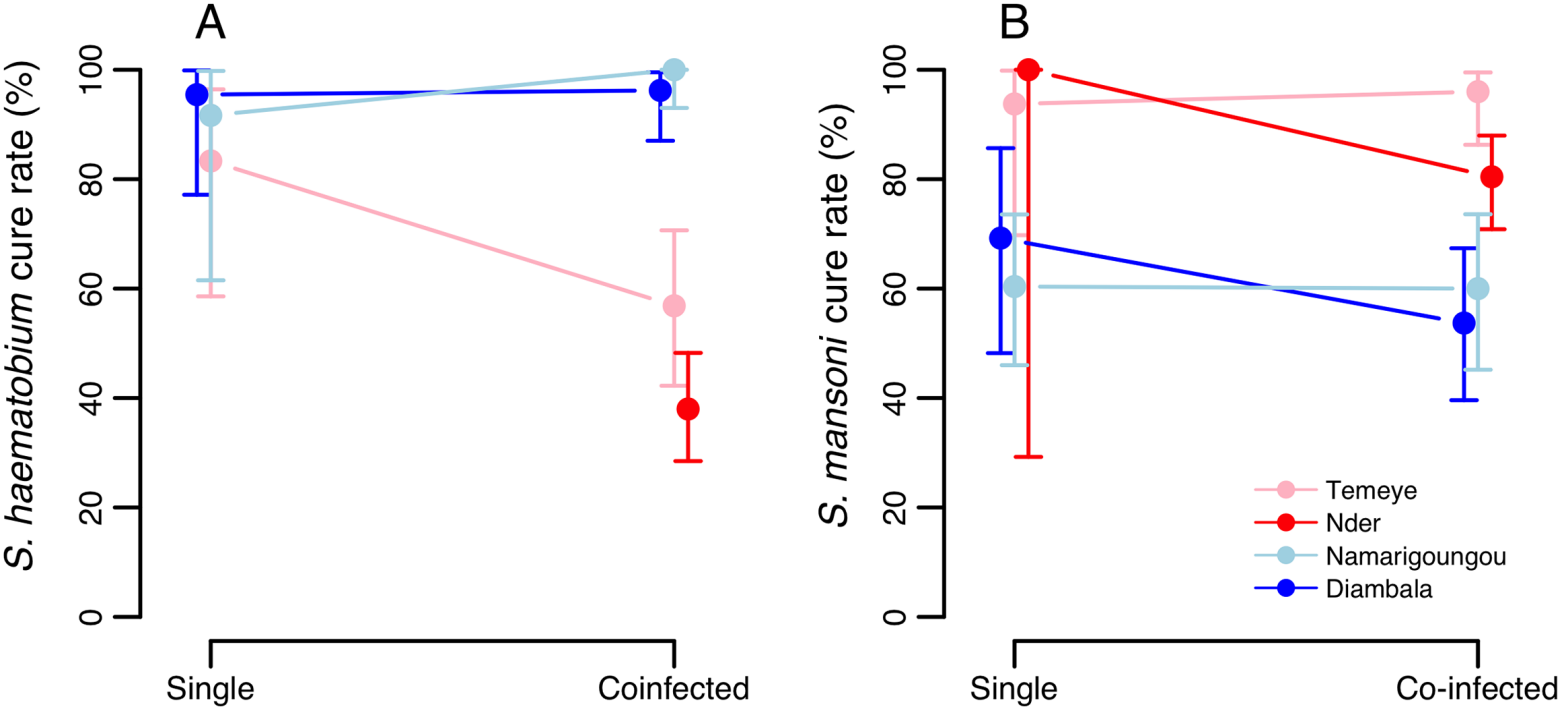
Parasitological cure rates for (A) *S*. *haematobium* and (B) *S*. *mansoni* with respect to co-infection status, in four co-endemic villages in Senegal (red/pink) and in Niger (blue). Raw data are plotted with exact 95% confidence intervals.

Raw cure rates for each village and schistosome species according to co-infection status are shown in Fig 4. For *S*. *haematobium*, CR was high (>90%) in Niger and showed no clear association with co-infection status. In Senegal, CR for *S*. *haematobium* was lower, particularly in Nder (where all individuals were co-infected with *S*. *mansoni*) as well as in co-infected individuals in Temeye (Fig 4A, Table 5). In a model including data from all four villages in Senegal and Niger, co-infection did not significantly explain variation in S. *haematobium* clearance, when controlling for significant effects of village and initial *S*. *haematobium* infection intensity (Table 5A). In Temeye, co-infected children had a lower CR than those with single infections (Fig 4A). However, models showed that this effect was equally well explained by higher *S*. *haematobium* infection intensity as by co-infection. Considering data on parasitological cure from Temeye only, both co-infection status and initial intensity had equal explanatory power in univariate models (likelihood ratio tests for co-infection: χ^2^_1_ = 4.16, p = 0.041; log(eggs per 10ml): χ^2^_1_ = 4.15, p = 0.042). However, neither term added significant explanatory power when the other was already present in the model (likelihood ratio test for co-infection: χ^2^ = 1.74, p = 0.187; log(eggs per 10ml): χ^2^ = 1.74, p = 0.188), suggesting that these two variables strongly confound one another in Temeye.

**Table 5:** Factors predicting the probability of parasitological cure for (A) *S*. *haematobium*, (B) *S*. *mansoni* and (C) any schistosome infection, six weeks after PZQ treatment in Senegalese and Nigerien villages where both species are endemic. Parameter estimates (on the logit scale) are from binomial mixed models. χ^2^ and p values are from likelihood ratio tests comparing full models with and without the term in question. ‘ref’ indicates the reference level of each factor.

Variable	df	Parameter estimate (SE)		χ^2^	p
**(A) *S*. *haematobium* (n = 302)**					
(Intercept)			0.519 (0.075)		
log(*S*. *haematobium* eggs/10ml)	1		-0.043 (0.014)	9.213	0.0024
*S*. *mansoni* infection	3	Uninfected (ref)	0	1.101	0.7767
		Light	-0.032 (0.064)		
		Moderate	-0.038 (0.071)		
		Heavy	0.031 (0.093)		
Village	3	Nder (ref)	0	84.901	<0.0001
		Namarigoungou	0.543 (0.065)		
		Diambala	0.516 (0.062)		
		Temeye	0.244 (0.063)		
**(B) *S*. *mansoni* (n = 344)**					
(Intercept)			0.950 (0.110)		
log(*S*. *mansoni* epg)	1		-0.016 (0.020)	0.675	0.4112
*S*. *haematobium* infection	2	Uninfected (ref)	0	3.000	0.2232
		Light	-0.040 (0.056)		
		Heavy	-0.152 (0.089)		
Village	3	Nder (ref)	0	31.351	<0.0001
		Namarigoungou	0.250 (0.069)		
		Diambala	-0.262 (0.068)		
		Temeye	0.121 (0.074)		
**(C) Both species combined (n = 399)**		(Intercept)	-0.402 (0.314)		
Infection status	1	One species (ref)	0	3.066	0.0799
		Two species	-0.411 (0.235)		
Village	3	Nder (ref)	0	16.075	0.0011
		Namarigoungou	0.964 (0.313)		
		Diambala	0.962 (0.313)		
		Temeye	1.159 (0.322)		

For *S*. *mansoni*, CR was lower in Niger than in Senegal, though in neither country did CR depend strongly on co-infection status (Fig 4B). In a model including data from all four villages, co-infection did not significantly explain *S*. *mansoni* clearance probability, when controlling for other effects, particularly strong variation between the two countries (Table 5B). Among those who did not clear *S*. *mansoni* entirely, the number of remaining eggs was also not predicted by *S*. *haematobium* co-infection (GLM on logged egg counts: co-infection term χ^2^ = 2.47, p = 0.116). In a model where clearance of all schistosome eggs of both species six weeks after PZQ was the response, there was marginal evidence that co-infected individuals were less likely to clear all parasites than those with single species infections, when controlling for differences in clearance rates between villages (Table 5C).

### *S*. *haematobium* longitudinal infection dynamics

**Fig 5:**
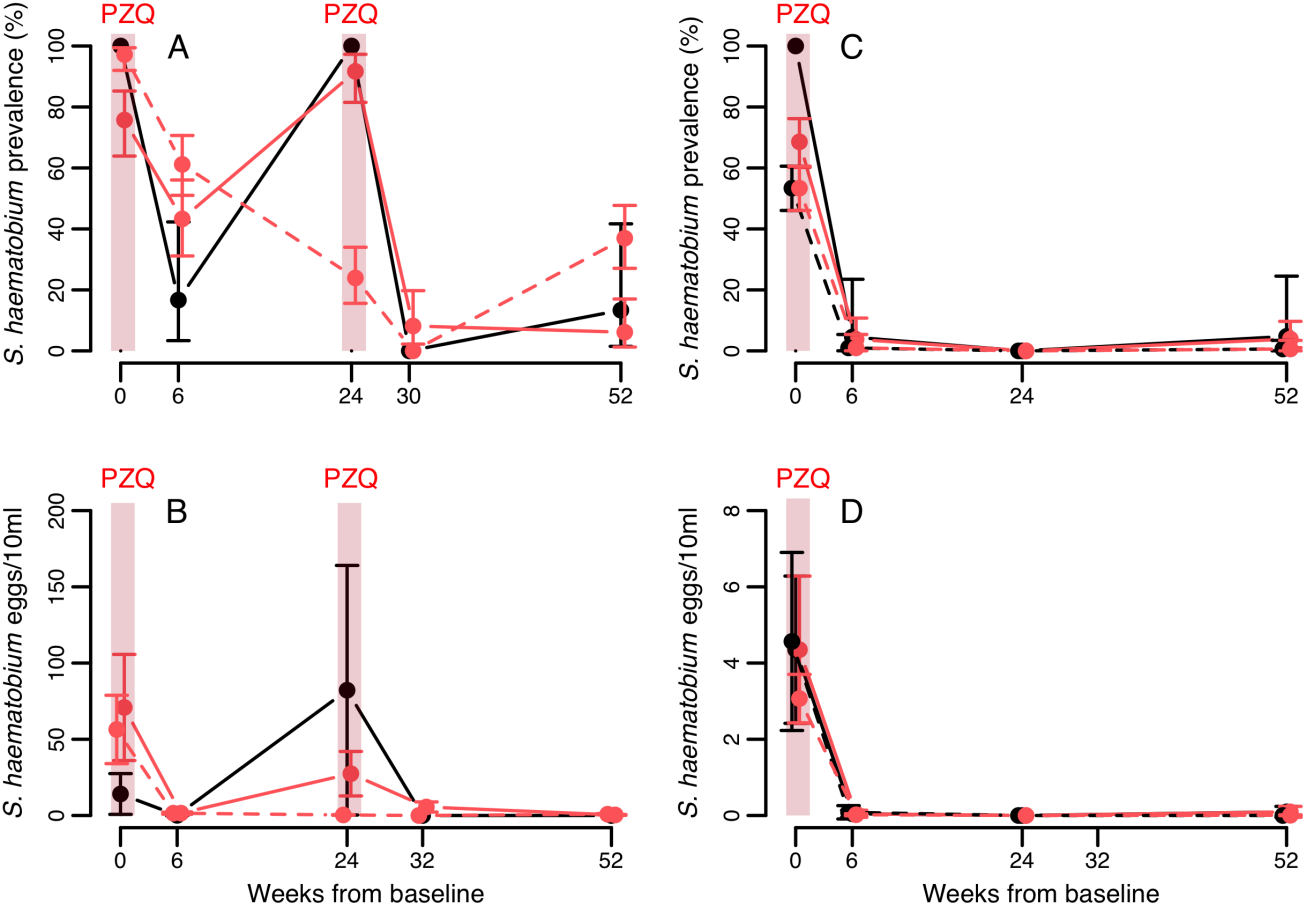
Longitudinal dynamics of *S*. *haematobium* in Senegal (A,B) and Niger (C,D), according to co-infection status with *S*. *mansoni* at baseline. Means from raw data are plotted with 95% confidence intervals. Confidence intervals for prevalence are exact, and for intensity data were calculated using a negative binomial GLM on egg counts including zero counts. Black and red lines indicate baseline single infections and co-infections with *S*. *mansoni* respectively. Solid and dashed lines represent the villages of Temeye and Nder in A and B, and Diambala and Namarigoungou in C and D respectively. There is no line for single infections in Nder because all infections in that village were co-infections.

**Fig 6:**
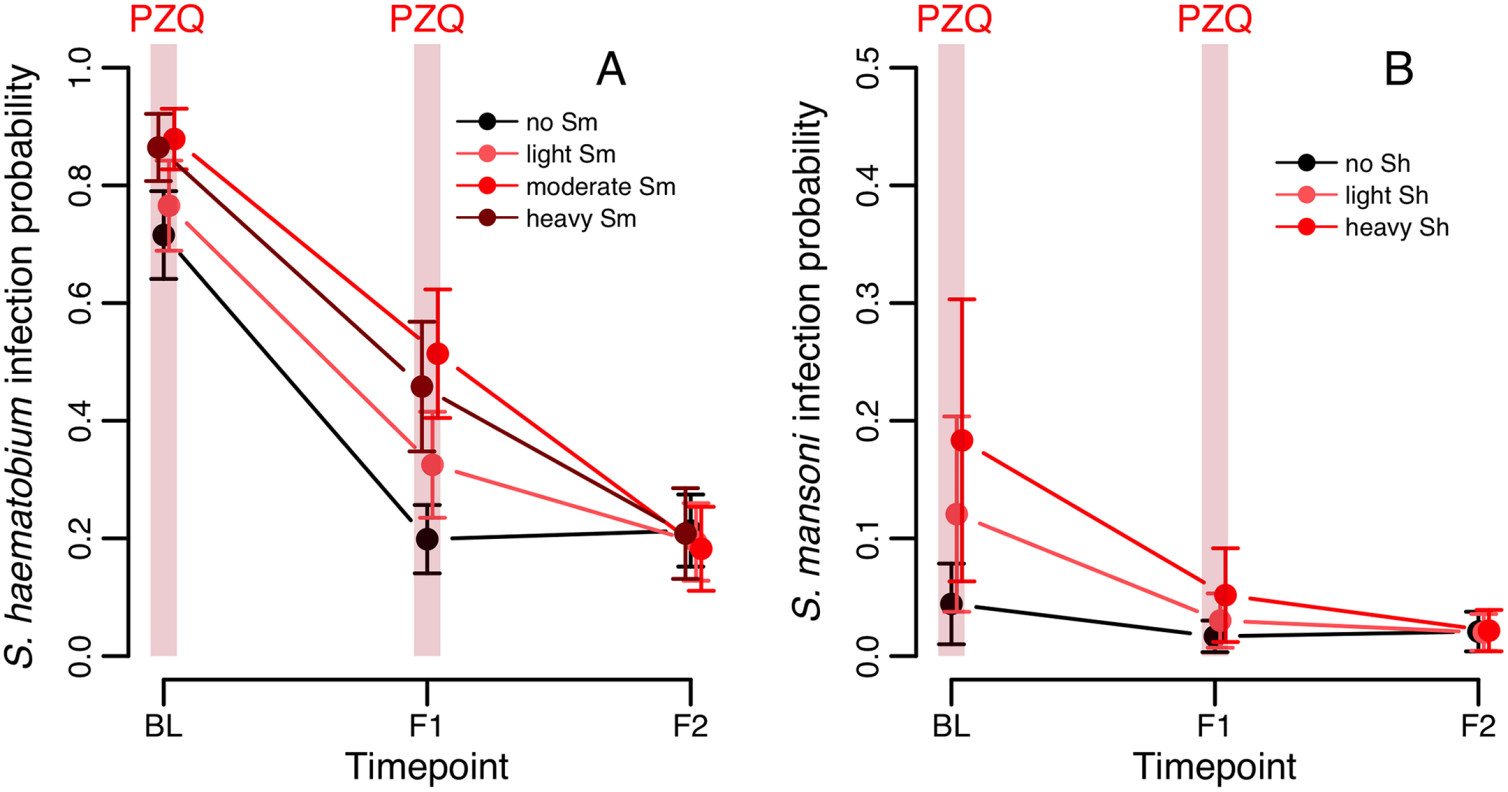
Predicted changes in the probability of (A) *S*. *haematobium* and (B) *S*. *mansoni* infection, over successive rounds of treatment in the Malian cohort according to co-infection status at baseline. Values shown are (cluster-specific) predicted values and standard errors from a binomial mixed model, for a child of average age in the dataset. BL = baseline, F1 = first annual follow-up1, F2 = second annual follow-up.

Patterns of *S*. *haematobium* infection post-treatment varied widely across sites. In Senegal, rapid re-infection with *S*. *haematobium* was seen in Temeye, though not in Nder (Fig 5A and 5B), whereas in both Nigerien villages *S*. *haematobium* re-infection was much slower (Fig 5C and 5D). In Mali, *S*. *haematobium* infection probability was higher in *S*. *mansoni* infected children at baseline, and declined less rapidly, though by the second annual follow-up, no differences in prevalence were apparent according to baseline *S*. *mansoni* status (Fig 6A; Table A in S1 Text). At the first annual follow-up (F1), an individual’s *S*. *haematobium* infection probability was predicted by their change in *S*. *mansoni* infection since baseline: while controlling for baseline *S*. *haematobium* status (i.e. predisposition to the focal species), children persistently infected with *S*. *mansoni* (at baseline and follow-up) were more likely to be infected by *S*. *haematobium* than those that never had *S*. *mansoni*, while those that either lost or gained *S*. *mansoni* since baseline had intermediate infection probabilities (*S*. *mansoni* change: χ^2^_3_ = 12.06, p = 0.007, controlling for significant effects of age, gender and baseline *S*. *haematobium* infection category; Fig B in S1 Text). Among the 2197 children recruited at the 20 schools where both schistosome species were monitored for all three years, 475 (22%) were only sampled at baseline, 466 (21%) were seen in two of the three years, and 1256 (57%) were followed-up in all three years. The mean number of follow-ups was 1.36. Drop-out analysis showed that the probability of children having full follow-up data (and therefore being included in these longitudinal analyses) was not affected by baseline infection status for *S*. *haematobium* or *S*. *mansoni*, but declined with age and differed across regions (Table B in S1 Text).

### *S*. *haematobium* re-infection isolated from lack of clearance

**Fig 7:**
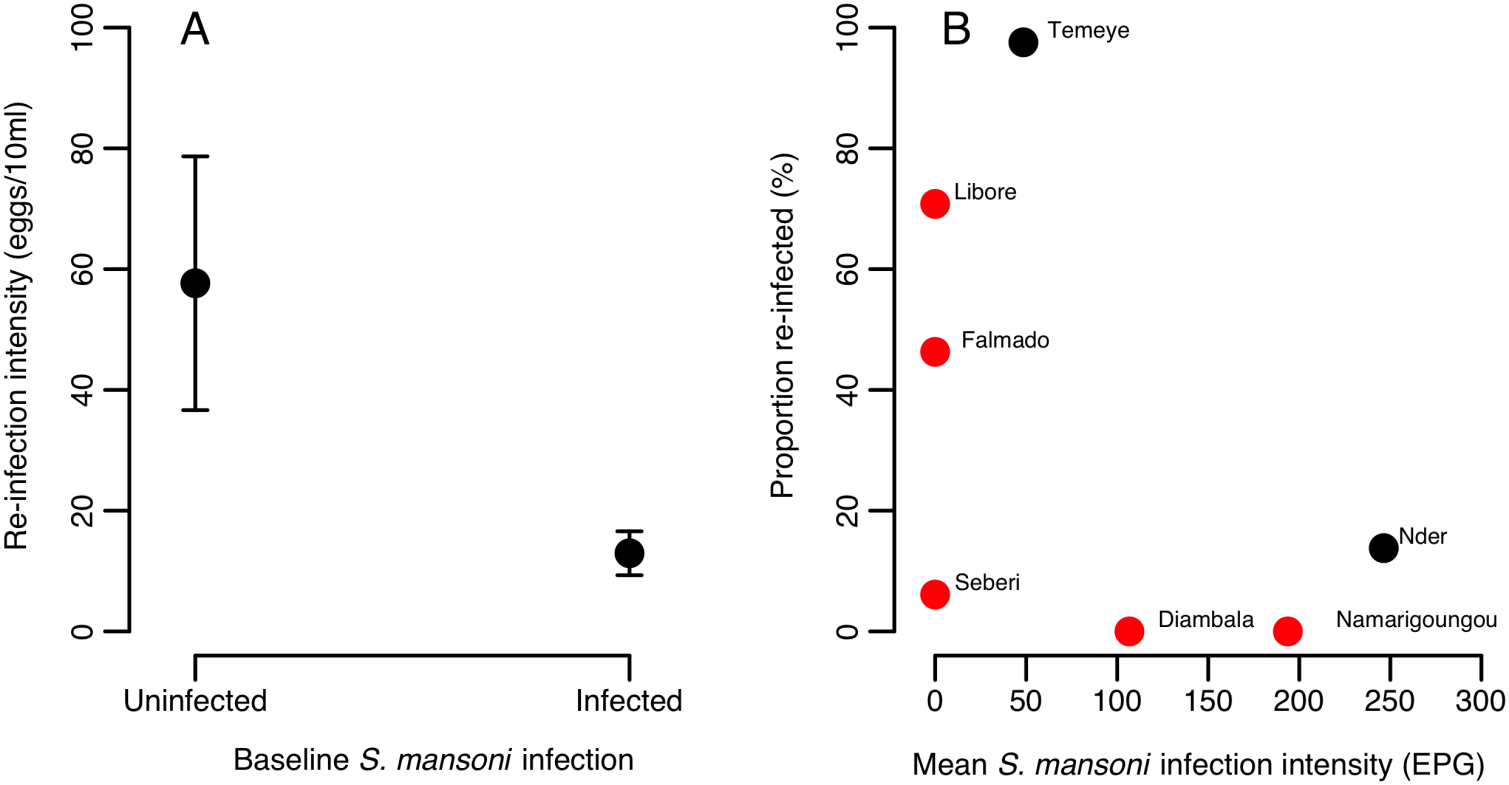
Relationship between S. haematobium re-infection 6 months post-PZQ in Senegalese and Nigerien villages, and S. mansoni co-infection at the point of treatment (baseline). (A) Negative association between *S*. *mansoni* co-infection and the intensity of *S*. *haematobium* re-infection from a negative binomial GLM on Senegalese data (n = 70). Values are predicted values from the minimal model in Table 6A, for an individual of average age, living in Temeye with a light *S*. *haematobium* infection prior to treatment. Errors bars represent 1 standard error. (B) Relationship between mean *S*. *mansoni* infection intensity and the proportion of individuals re-infected with *S*. *haematobium* six months after PZQ treatment across seven villages in Niger (red dots) and Senegal (black dots).

No *S*. *haematobium* re-infection was observed after 6 months in Nigerien co-endemic villages, so variation in *S*. *haematobium* re-infection was only examined in Senegal. Among individuals with *S*. *haematobium* at baseline that had cleared infection six weeks after PZQ treatment, six month re-infection rates were 14% in Nder (where 100% children had *S*. *mansoni* at baseline), and 98% in Temeye (96% in children co-infected with *S*. *mansoni* at baseline vs. 100% in children singly infected). In a model including data from both villages, re-infection probability was strongly predicted by village (higher in Temeye than Nder, χ^2^_1_ = 133.71, p<0.001) and was also higher in individuals with heavy compared to light *S*. *haematobium* infection at baseline (χ^2^_2_ = 6.17, p = 0.0130). As expected given the limited variation in re-infection rates in Temeye (the only village where co-infection showed some variation), no significant effect of baseline *S*. *mansoni* co-infection on re-infection probability was detected while controlling for village and other effects (χ^2^_1_ = 1.27, p = 0.259). However, across both Senegalese villages, the intensity of *S*. *haematobium* re-infection at six months post-treatment (which varied more widely, range 0–372 eggs/10ml), was significantly lower in those individuals co-infected by *S*. *mansoni* at baseline (Table 6A, Fig 7A). The negative binomial model provided a good fit to re-infection intensity data, with observed counts closely matching expected values (Fig C in S1 Text) and a low overdispersion parameter (α = 1.2). It was not possible to test for an interaction between co-infection status and village, as all individuals in Nder were co-infected at baseline. However, when limiting the analysis to the village of Temeye, the same negative effect of *S*. *mansoni* co-infection on *S*. *haematobium* re-infection intensity was seen (χ^2^ = 12.02, p = 0.0005). To explore this effect further, we tested how the change in an individual’s *S*. *mansoni* infection status between baseline, 6 weeks and 6 months predicted re-infection intensity, using data from both villages. This showed that the major difference in *S*. *haematobium* re-infection intensity was between those individuals that were *S*. *mansoni* negative at baseline but gained infection by 6 months (pattern 0-0-1), compared to those that were cleared of their original *S*. *mansoni* infection (1-0-0 and 1-0-1; Table C in S1 Text). We also examined geographical variation in *S*. *haematobium* re-infection rates across all of the seven villages in Senegal and Niger assessed in our previous studies [4, 5]. We noted that village-level *S*. *haematobium* re-infection rate at six months tended to decrease with increasing baseline mean *S*. *mansoni* infection intensity (Fig 7B).

**Table 6:** Re-infection intensity 6-months after baseline among individuals that cleared their infection 6 weeks after PZQ treatment, modelled by negative binomial GLMs. Baseline infection intensity for the focal species was retained in models even when not significant, so the effect of co-infection over and above effects of the focal species could be assessed. χ^2^ and p values are from likelihood ratio tests comparing full models with and without the term in question. Age was mean-centred in analyses. ‘ref’ indicates the reference level of each factor.

Variable	df	Parameter estimate (SE)		χ^2^	p
**S. haematobium eggs/10ml (n = 70; Senegal only)**					
(Intercept)					
Baseline *S*. *haematobium* infection	1	Light (ref)	0	1.891	0.1691
		Heavy	0.800 (0.556)		
Sex	1	Male (ref)	0	0.013	0.9092
		Female	0.051 (0.429)		
Age	1		-0.189 (0.100)	4.322	0.0376
Age^2^	1		-0.041 (0.037)	1.15	0.2836
Village	1	Nder (ref)	0	50.374	<0.0001
		Temeye	4.396 (0.720)		
Baseline *S*. *mansoni* infection	1	Not infected (ref)	0	10.713	0.0011
		Co-infected	-1.657 (0.498)		
**S. mansoni eggs/gram(n = 225, Senegal and Niger)**					
(Intercept)			6.598 (0.393)		
Baseline *S*. *mansoni* infection	2	Light (ref)	0	2.798	0.2469
		Moderate	0.440 (0.249)		
		Heavy	0.092 (0.363)		
Sex	1	Male (ref)	0	0.296	0.5866
		Female	0.119 (0.220)		
Age	1		-0.021 (0.042)		
Age^2^	1		-0.039 (0.014)	6.818	0.0090
Village	3	Nder (ref)	0	78.815	<0.0001
		Temeye	-0.902 (0.322)		
		Diambala	-3.608 (0.353)		
		Namarigoungou	-2.255 (0.343)		
Baseline *S*. *haematobium* infection	1	Not infected (ref)	0	4.683	0.0305
		Co-infected	-0.602 (0.266)		

### *S*. *mansoni* longitudinal patterns

**Fig 8:**
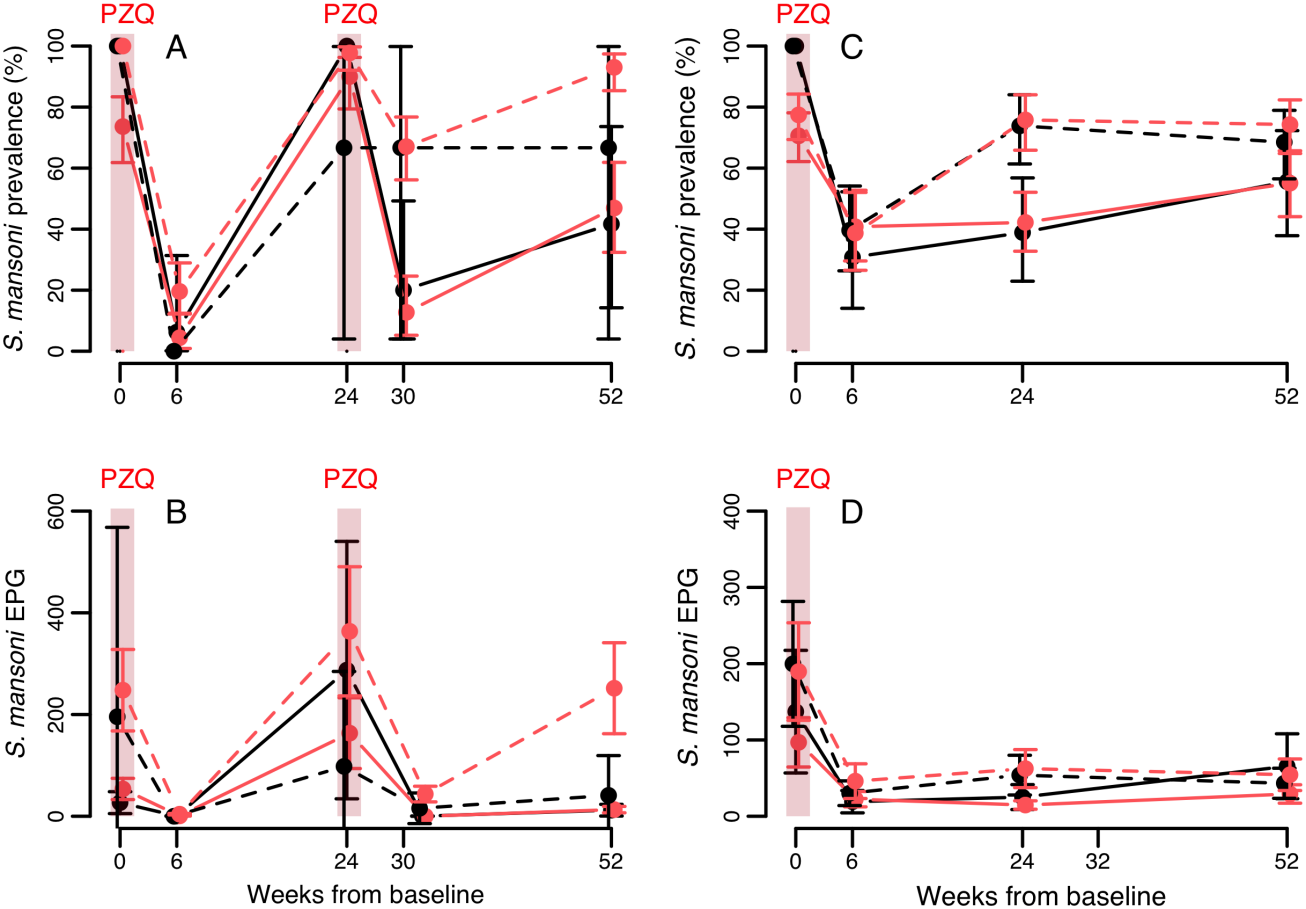
Longitudinal dynamics of *S*. *mansoni* in Senegal (A, B) and Niger (C, D), according to co-infection status at baseline. Means from raw data are plotted with 95% confidence intervals. Confidence intervals for prevalence are exact, and for intensity data were calculated using a negative binomial GLM on egg counts including zero counts. Black and red lines indicate baseline single infections and co-infections with *S*. *haematobium* respectively. Solid and dashed lines represent the villages of Temeye and Nder in A and B, and Diambala and Namarigoungou in C and D respectively. There is no line for single infections in Nder because all infections in that village were co-infections.

Similar to *S*. *haematobium*, re-infection for *S*. *mansoni* was much quicker in Senegalese than Nigerien villages (Fig 8). In Mali, *S*. *mansoni* prevalence declined over two annual PZQ treatments, and this pattern differed only a minor amount according to baseline *S*. *mansoni* infection status (Table B in S1 Text): *S*. *mansoni* infection probability was higher in *S*. *haematobium* positive children at baseline and declined less rapidly, though no differences according to baseline *S*. *haematobium* infection status were visible after two years (Fig 7B). After one year, similar to the pattern seen for *S*. *haematobium*, *S*. *mansoni* infection probability was weakly predicted by changes in *S*. *haematobium* infection: children persistently infected with *S*. *haematobium* (at both baseline and first follow-up) were more likely to be infected with *S*. *mansoni* than those that never had *S*. *haematobium*, with those that either lost or gained *S*. *haematobium* intermediate (*S*. *haematobium* change: χ^2^_3_ = 10.22, p = 0.017, controlling for effects of age, gender, region and baseline *S*. *mansoni* infection category; Fig B in S1 Text).

### *S*. *mansoni* re-infection isolated from lack of clearance

In the four Senegalese and Nigerien villages, among children that had *S*. *mansoni* at baseline but cleared it 6 weeks post-PZQ, re-infection probability at six months was strongly predicted by village (χ^2^_3_ = 65.73, p<0.0001), largely reflecting differences between the two countries (Fig 8). *S*. *haematobium* co-infection status did not predict *S*. *mansoni* re-infection probability (χ^2^_1_ = 2.312, p = 0.128), while controlling for village, baseline *S*. *mansoni* infection intensity category (χ^2^_2_ = 0.799, p = 0.671), and age (Age^2^ χ^2^_1_ = 4.883, p = 0.027). These results reflect inconsistent patterns of re-infection rate according to *S*. *haematobium* co-infection status across the four villages (single vs. co-infected in Temeye, Nder, Diambala and Namarigoungou: 100% vs. 88%, 67% vs. 97%, 33% vs. 38% and 92% vs. 59% respectively). In models of *S*. *mansoni* re-infection intensity, again the strongest effect was village (Table 6B). However, a weak main effect of *S*. *haematobium* co-infection on re-infection intensity was also detected while controlling for covariates, indicating slightly lighter *S*. *mansoni* re-infection overall among individuals co-infected with *S*. *haematobium* at baseline (Table 6B). When an interaction term between village and co-infection status was included in the model, however, this was also weakly significant (χ^2^_3_ = 7.943, p = 0.047), indicating that the effect of co-infection differed somewhat across sites. This interaction term reflected a weak positive association between co-infection and *S*. *mansoni* re-infection intensity in Nder and Diambala compared to a weak negative association in Temeye and Namarigoungou (Fig D in S1 Text), though in single village analyses, the co-infection term was always non-significant (all p>0.08). Overall therefore, *S*. *mansoni* re-infection was not strongly predicted by initial *S*. *haematobium* co-infection status, and the relatively weak negative effect of co-infection on egg counts at six months was not consistent across sites. We also checked whether any potential effect of *S*. *haematobium* co-infection on re-infection intensity might depend on an individual’s initial *S*. *mansoni* infection intensity, but there was no evidence for such an interaction (χ^2^_2_ = 3.451, p = 0.178).

## Discussion

Despite frequent co-endemicity of *S*. *haematobium* and *S*. *mansoni* across Africa, little is known about the potential for within-host interactions between these two species, or whether such interactions might affect the impact of PZQ treatment or host immunity. Here, we analyse epidemiological data from school-age children in three West African countries (Senegal, Niger and Mali), to explore whether these two species may interact with one another in the context of PZQ treatment.

At baseline, associations between urogenital and intestinal schistosomiasis were generally positive in all three countries: for both *S*. *haematobium* and *S*. *mansoni*, infection intensity increased with the degree of co-infection, while controlling for other effects such as age, gender and location, and in Mali children with one species were more likely to be infected by the other, with some geographic variation in the strength of this association. Similar positive associations prior to treatment have been previously reported from other co-endemic villages in Senegal [7], and Cameroon [38]. Such positive associations may be driven by a number of processes, including correlated exposure of children to infective *Bulinus* and *Biomphalaria* snails at the same transmission sites, correlated immune susceptibility of individuals to infection by the two species, and/or facilitative interspecific interactions within the host. Few studies have examined the evidence for correlated exposure to *S*. *haematobium* and *S*. *mansoni*, and while some have in fact suggested these species may have distinct local transmission sites within villages [39, 40], further work is needed to examine the extent to which correlated exposure, or other mechanisms, may drive the positive associations between schistosome species documented here and elsewhere.

In analysing data from two efficacy studies with similar methodology in Senegal and Niger, we found that the efficacy (ERR) of two (three week spaced) PZQ doses against either *S*. *haematobium* or *S*. *mansoni* was not significantly altered by the presence of co-infection. This is encouraging, though the effect of co-infection on efficacy still requires testing for the standard, single 40mg/kg dose of PZQ widely used in control programmes. Parasitological cure rates told a somewhat different story, with clearance probability for *S*. *haematobium* reduced in mixed infections compared to single infections in Senegal, but not Niger. Further analysis showed that this effect of *S*. *mansoni* on *S*. *haematobium* clearance disappeared once initial infection intensity was controlled for, suggesting intensity differences likely underpinned reduced clearance of *S*. *haematobium* from mixed infections in Senegal. While it is well known that heavy infections are less likely to be cleared by a standard dose of PZQ [41–43], a key question arising from our findings is to what extent *S*. *mansoni* co-infection causes, or is merely correlated with, increased infection intensity for *S*. *haematobium*. The answer will determine whether co-endemicity in an area per se is cause to consider higher or more repeated doses of PZQ to get full infection clearance. Our finding of reduced overall clearance for *S*. *haematobium*/*S*. *mansoni* co-infections compared to single infections agrees with a recent meta-analysis of studies using the standard 40mg/kg PZQ dose [44] in suggesting mixed infections may be harder to clear. Again whether this observation is due to mixed infections simply being heavier, or other effects remains unclear. Our results did not suggest a consistent difference in PZQ efficacy against the two species: *S*. *mansoni* showed lower ERR and CR than *S*. *haematobium* in Niger [4], though this was not seen in Senegal [5]. Another study with very similar methodology reported higher ERR and CR for *S*. *mansoni* than *S*. *haematobium* in co-endemic sites in Cameroon [45]. These mixed findings echo two recent studies synthesizing cure rate data for urogenital and intestinal schistosomiasis, which showed large geographic variability but only limited species differences in PZQ-related clearance overall [43, 44]. We also show that even after controlling for differences in initial infection intensity, co-infection status and other variables, large differences in cure rates were observed between Senegal and Niger for both urogenital and intestinal schistosomiasis. Moreover, while *S*. *haematobium* ERR was similar in the Senegalese and Nigerien villages, ERR for *S*. *mansoni* was notably lower in Niger. These geographic differences may reflect intraspecific genetic differences in schistosomes, as seen across other West African countries [46], or geographic differences in the prevalence of cryptic hybrid schistosomes that may differ in susceptibility to PZQ [47], both possibilities warranting further investigation.

Patterns of re-infection for both schistosome species varied markedly between countries, and to a lesser extent among sites within each country. Variation in transmission site ecology, for example changes in the abundance of *Biomphalaria* and *Bulinus* snails, as well as seasonality are likely to play important roles in driving these differences [48]. However, when controlling for geographic location, we revealed a somewhat counterintuitive effect of interspecific co-infection on the degree of short-term re-infection (6 months after PZQ treatment). Since baseline associations between *S*. *haematobium* and *S*. *mansoni* were generally positive, one might expect to see higher levels of re-infection among individuals that were co-infected at baseline, that would re-establish these positive associations post-treatment. However, we found the opposite pattern in data from Senegal. Despite *S*. *mansoni* co-infected individuals having higher *S*. *haematobium* egg counts at baseline, after effective treatment they had significantly lower *S*. *haematobium* egg counts six months later than those with single infections at baseline. This result could indicate some kind of asymmetric competitive interaction between *S*. *mansoni* and *S*. *haematobium*, whereby *S*. *mansoni* infection at the point of treatment limits the extent to which *S*. *haematobium* can re-infect or shed eggs after treatment. Although it was not possible to test for the same individual-level effect in Niger (since no participants were re-infected by *S*. *haematobium* at six months), we did observe at a population-level that the proportion of individuals re-infected with *S*. *haematobium* in a given location was negatively related to baseline mean intensity of *S*. *mansoni* infection. This pattern that is at least consistent with the effect seen in Senegal, though may be influenced by other factors varying across sites, such as environmental factors related to snail ecology. While there was some evidence for the reverse pattern—*S*. *haematobium* co-infection at baseline limiting *S*. *mansoni* re-infection, the effect was much weaker and geographically variable. Interestingly, a study with similar methodology in Cameroon (conducted under the same multi-country CONTRAST project as the Senegal and Niger studies reported here) also showed a reduced probability of *S*. *haematobium* re-infection among children co-infected by *S*. *mansoni* at the time of PZQ treatment, though the statistical significance of this difference was not tested [45]. Further studies are now needed to test the generality of these patterns across a range of other co-endemic settings.

If these patterns do reflect asymmetric competition between *S*. *mansoni* and *S*. *haematobium* after treatment, a range of possible mechanisms could be responsible. First, if baseline *S*. *mansoni* co-infection is associated with faster or more intense *S*. *mansoni* re-infection, our findings could arise though interspecific competition during re-infection. Such competition could occur in the currency of mates, nutritional resources like blood (an important food source for adult schistosomes) [49], or immune-mediated competition via cross-immunity to cercariae, immature or adult worms. Mate competition between *S*. *mansoni* and *S*. *haematobium* has been previously documented [14–16], and competitive asymmetry could arise if *S*. *mansoni* males divert *S*. *haematobium* females away from their preferred ovipositing site more often than the reverse pattern [16]. Competition for resources among schistosome species may also affect patterns detected here, though to our knowledge resource-based competition among schistosomes has not yet been explored. It also remains unclear to what extent *S*. *mansoni* and *S*. *haematobium* elicit cross-protective immune responses in humans. The idea of heterologous immunity in schistosomes has a long history, and there is clear evidence from animal models that challenge by one species can sometimes protect against, or at least limit egg output from, a challenge infection by another species [50]. Challenge experiments in hamsters have shown that *S*. *mansoni* stimulates stronger cross protection than *S*. *haematobium* [20], and that *S*. *mansoni* competes with *S*. *haematobium* at several stages, impairing worm development, sexual maturation and egg shedding [22]. Moreover, a recent study conducted close to our study sites in Senegal [9] provided preliminary evidence for cross-immunity between these two species in humans, as measured by serum cytokine production in response to egg or adult worm antigen. Intriguingly, this study also showed that *S*. *mansoni* elicited somewhat stronger heterologous responses than *S*. *haematobium* [9], consistent with our finding that *S*. *mansoni* was associated with reduced *S*. *haematobium* re-infection, but not necessarily the other way around. Thus cross-immunity between *S*. *mansoni* and *S*. *haematobium* may well occur, and its importance in humans warrants further investigation. To try and distinguish between mate, resource and immune-mediated competition in our analyses, we tested whether the effect of baseline *S*. *mansoni* status on *S*. *haematobium* re-infection varied according to subsequent *S*. *mansoni* clearance or re-infection. Mate competition would be expected to show the strongest effect in individuals that did not fully clear *S*. *mansoni* upon treatment, and cross-reactive immune responses against re-infecting *S*. *mansoni* would predict the strongest effect in children who were re-infected with *S*. *mansoni* six months later, compared to those with no patent infection. In fact, we found that effect of baseline *S*. *mansoni* infection was similar regardless of whether individuals were re-infected by S. *mansoni* six months after treatment, and was weakest in those that did not clear *S*. *mansoni* in the first place. This suggests that the legacy of *S*. *mansoni* infection, rather than active infection, is enough to generate competition with re-infecting *S*. *haematobium*, which is possible if PZQ treatment exposes *S*. *mansoni* antigen that elicit heterologous immunity. Indeed, PZQ treatment is known to alter immunity to schistosomes [51], an effect that could extend to heterologous immunity. While we only examined school-age children here, immunologically-driven patterns may differ depending on the length of exposure to schistosomes. Therefore, studies involving adults would also be useful to further explore the patterns reported here.

**Fig 9:**
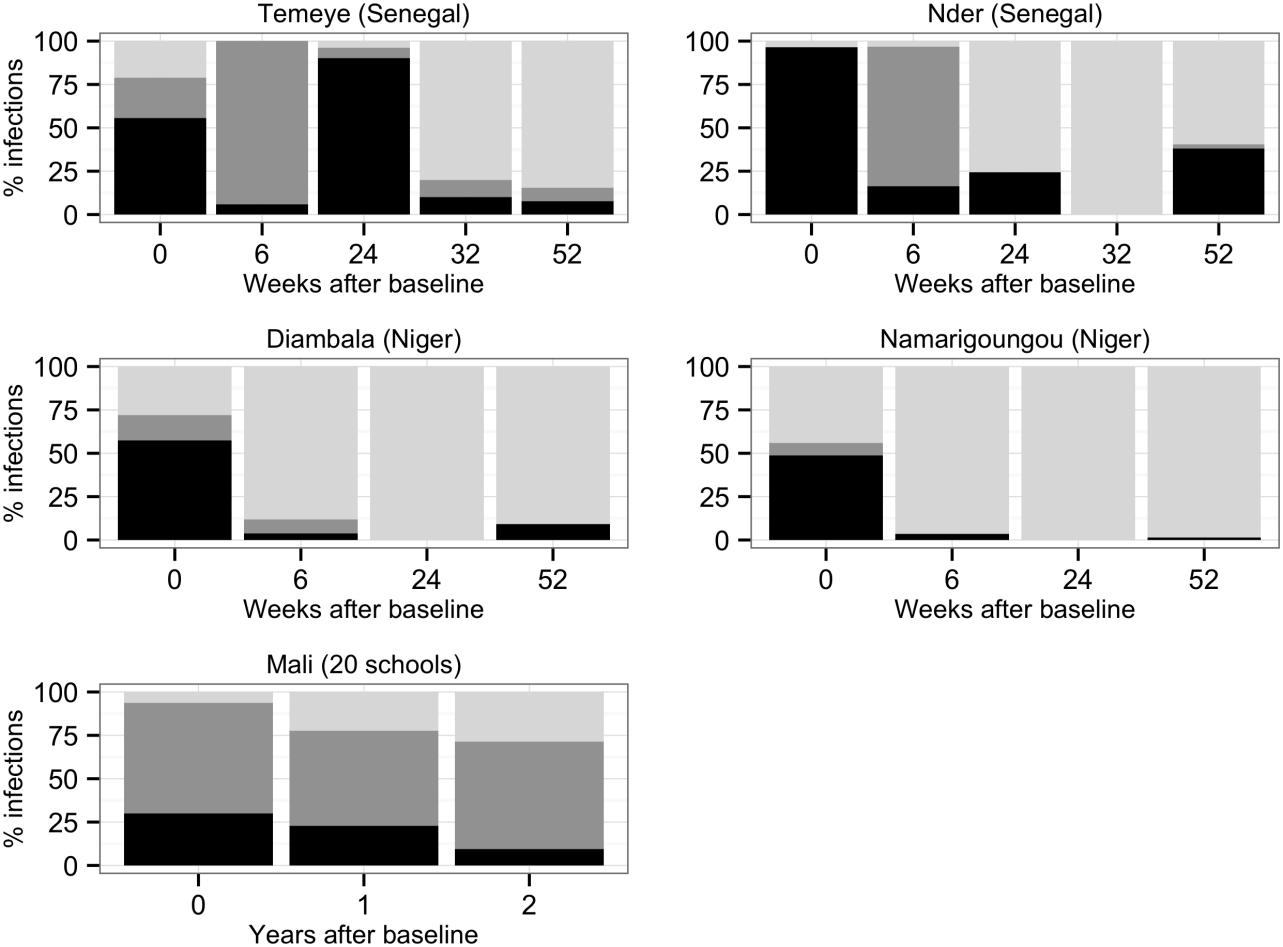
Changes in the proportion of schistosome infections at each follow-up point that were attributable to *S*. *mansoni* alone (pale grey), *S*. *haematobium* alone (mid grey) or both species (co-infections, in black). Only children that were positive at baseline, and monitored at all follow-up occasions are included.

In apparent contrast to findings from Senegal, at co-endemic sentinel sites in Mali, the children most likely to have *S*. *haematobium* at the first annual follow-up were those that maintained *S*. *mansoni* infection over the same period. Children maintaining *S*. *haematobium* were also more likely to have *S*. *mansoni* at first follow-up. Despite appearing contradictory to findings from the Senegalese and Nigerien villages (where negative associations between co-infection and re-infection were seen), such positive correlations in Mali may simply reflect a lack of treatment in certain individuals: maintenance of infection with a given species could well indicate that these children missed PZQ treatment in the national treatment campaign, and therefore would also be more likely to harbor the other schistosome species at follow-up. While data from sentinel sites (where treatment coverage is imperfect), are less powerful than deliberate treatment follow-up studies for examining potential interactions between schistosome species, they can contribute to our understanding of how schistosome species composition is likely to change as preventative chemotherapy programmes advance. In this respect, it is noteworthy that in all three co-endemic West African countries examined here, among children that were infected at baseline and had complete follow-up, over time the proportion of schistosome infections attributable to *S*. *mansoni* as compared to *S*. *haematobium*, increased (Fig 9). A whole range of processes may drive such population-level trends, including altered exposure via changes in the relative abundance of *Biomphalaria* and *Bulinus* snails, or the re-infection effect we observed in Senegal, whereby *S*. *mansoni* infection appeared to limit *S*. *haematobium* re-infection after treatment. Whatever the underlying causes, it will be useful to study whether an increase in the contribution of *S*. *mansoni* to infections post-treatment is a general pattern beyond the three countries examined here.

In conclusion, our multi-country analysis of associations between *S*. *haematobium* and *S*. *mansoni* throughout the process of PZQ treatment and re-infection provided no evidence for altered efficacy of double-dose PZQ in co-infections compared to single infections, though co-infections were sometimes heavier and less likely to clear completely after treatment. We also show that while controlling for other effects, children co-infected with *S*. *mansoni* in Senegal showed reduced *S*. *haematobium* re-infection compared to singly infected children, which we suggest may be driven by treatment-induced antigen exposure and stimulation of cross-protective immune responses. Furthermore, although the temporal dynamics of prevalence and intensity for these two species were most strongly affected by location, suggesting local environmental factors are the dominant drivers of re-infection patterns, in all three co-endemic countries examined the proportion of infections attributable to *S*. *mansoni* relative to *S*. *haematobium* increased over time post-treatment. Studies in other co-endemic areas are warranted to test the generality of these findings, which have implications for our understanding of basic schistosome biology, as well as relevance for schistosomiasis control as we move towards elimination in mixed species settings.

## Supporting Information

S1 Text Figure A. Baseline patterns of schistosome infection intensity according to co-infection status in individual Malian schools. Figure B. Infection probability among Malian children in year F1 according to changes in co-infection status from baseline. Figure C. Observed and expected distribution of *S*. *haematobium* egg counts under NB2 model. Figure D. Variable relationship between *S*. *mansoni* re-infection intensity and co-infection across sites in Senegal and Niger. Table A. Model results on changes in infection probability over time in Malian children, according to baseline co-infection status. Table B. Predictors of follow-up in Malian cohort. Table C. Model results on how *S*. *haematobium* re-infection intensity in Senegal was predicted by changes in *S*. *mansoni* infection from baseline.
(DOCX)

S1 Checklist STROBE Checklist. (DOC)
