# Supplementary material for: Epidemiological Interactions between Urogenital and Intestinal Human Schistosomiasis in the Context of Praziquantel Treatment across Three West African Countries
Source: PLoS Negl Trop Dis. 2015 Oct 15;9(10):e0004019. doi: 10.1371/journal.pntd.0004019 (PMC4607489; doi:10.1371/journal.pntd.0004019)
Supplement: S1 Text — Figure A. Baseline patterns of schistosome infection intensity according to co-infection status in individual Malian schools. Figure B. Infection probability among Malian children in year F1 according to changes in co-infection status from baseline. Figure C. Observed and expected distribution of S. haematobium egg counts under NB2 model. Figure D. Variable relationship between S. mansoni re-infection intensity and co-infection across sites in Senegal and Niger. Table A. Model results on changes in infection probability over time in Malian children, according to baseline co-infection status. Table B. Predictors of follow-up in Malian cohort. Table C. Model results on how S. haematobium re-infection intensity in Senegal was predicted by changes in S. mansoni infection from baseline. (DOCX) [file pntd.0004019.s001.docx]

**Supplementary Figures and Tables**

**Figure A:** Baseline patterns of (A) *S. haematobium* and (B) *S. mansoni* infection intensity according to co-infection with the other schistosome species across 29 co-endemic schools in 3 regions of Mali. Each line represents a school, and colours show regions (pink=Bamako, green=Koulikoro, blue= Ségou).


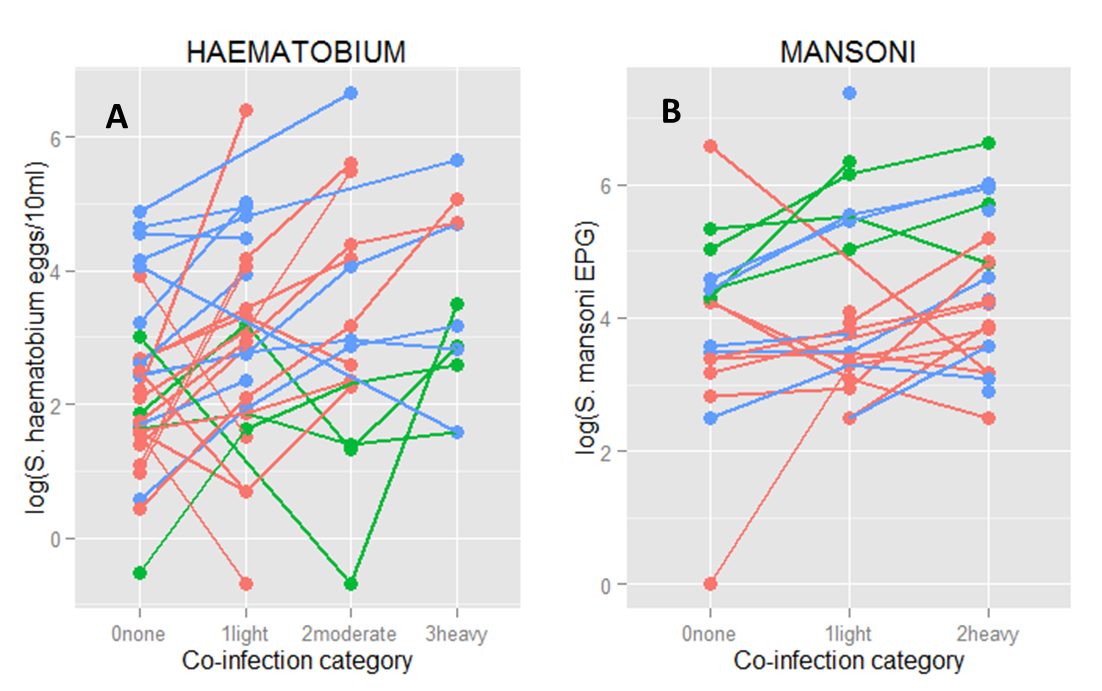


**Figure B:** Differences in infection probability one year from baseline in Malian school children, according to changes in co-infection status, for (A) *S. haematobium* (B) *S. mansoni*.


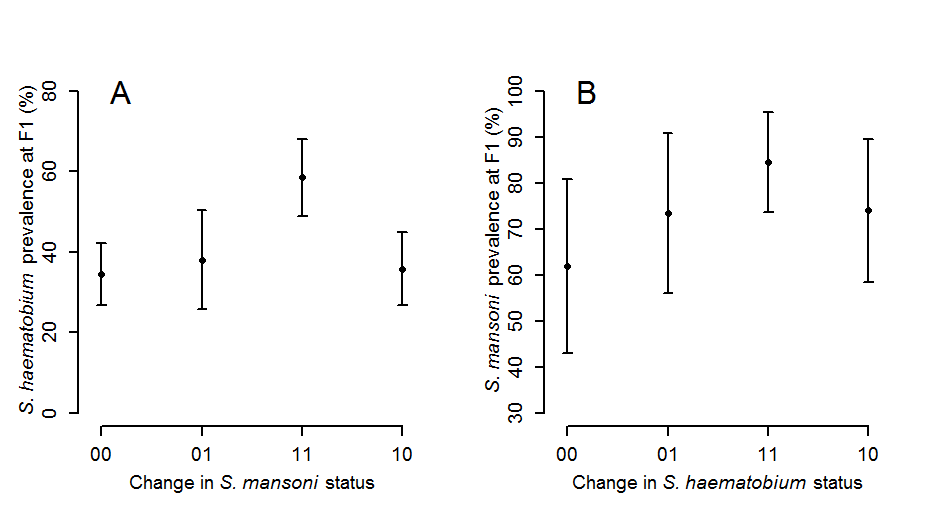


**Figure C:** Observed distribution of *S. haematobium* egg counts six months after PZQ treatment compared to that expected with a NB2 negative binomial distribution. Plot was produced using the COUNT package in R.


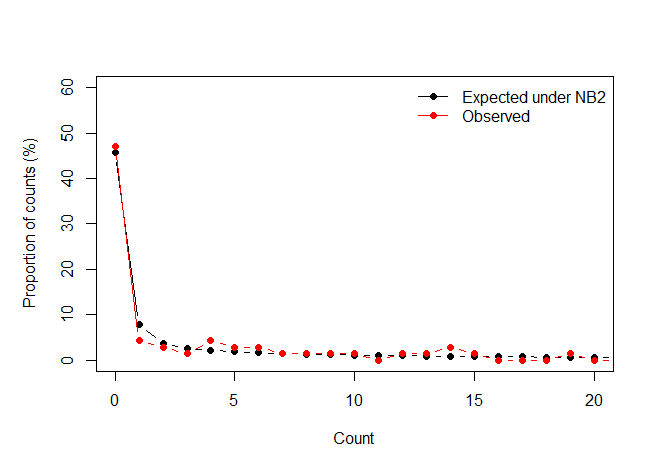


**Figure D:** Variable relationship between *S. mansoni* re-infection intensity 6 months post-PZQ and *S. haematobium* co-infection at the point of treatment (baseline), across Senegalese and Nigerien villages: Values are predicted values from a negative binomial model including a village by co-infection status interaction, for an individual of average age. Errors bars represent 1 standard error.

**
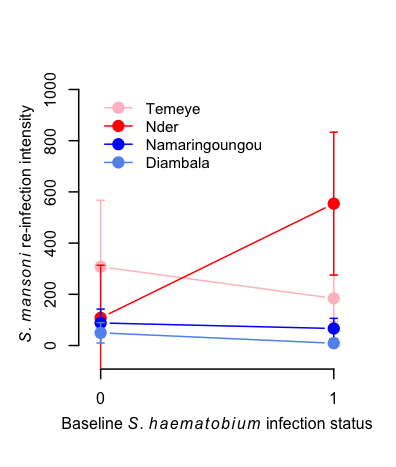
**

**Table A**: Results of models assessing changes in (A) *S. haematobium* and (B) *S. mansoni* infection probability over time in a cohort of Malian children under a national schistosomiasis control programme, as a function of baseline co-infection status and covariates. Parameter estimates (on the logit scale) are from binomial mixed models. χ^2^ and p values are from likelihood ratio tests comparing models with and without the term in question. Age was mean-centred in both analyses. ‘ref’ indicates the reference level of each factor.

| **Variable** | **df** | **Parasite estimate (SE)** | | χ^2^ | **p** |
| --- | --- | --- | --- | --- | --- |
| **(A) *S. haematobium*** |  |  |  |  |  |
| (Intercept) |  |  | 0.842 (0.363) |  |  |
| Region | 2 | Bamako (ref) | 0 | 10.189 | 0.0061 |
|  |  | Koulikoro | -0.742 (0.614) |  |  |
|  |  | Ségou | 1.275 (0.491) |  |  |
| Year | 2 | Baseline (ref) | 0 |  |  |
|  |  | F1 | -2.318 (0.160) |  |  |
|  |  | F2 | -2.228 (0.166) |  |  |
| Baseline *S. mansoni* infection | 3 | Uninfected (ref) | 0 |  |  |
|  |  | Light | 0.260 (0.260) |  |  |
|  |  | Moderate | 1.058 (0.345) |  |  |
|  |  | Heavy | 0.930 (0.354) |  |  |
| Year*Baseline *S. mansoni* infection | 6 | Uninfected (ref) | 0 | 31.081 | <0.0001 |
|  |  | F1:Light | 0.404 (0.315) |  |  |
|  |  | F2:Light | -0.379 (0.328) |  |  |
|  |  | F1:Moderate | 0.393 (0.398) |  |  |
|  |  | F2:Moderate | -1.253 (0.444) |  |  |
|  |  | F1:Heavy | 0.297 (0.399) |  |  |
|  |  | F2:Heavy | -0.960 (0.423) |  |  |
| Age | 1 |  | -2.436 (2.915) |  |  |
| Age^2^ | 1 |  | -5.227 (2.657) | 3.837 | 0.050 |
| Sex | 1 | Male (ref) | 0 | 4.038 | 0.044 |
|  |  | Female | -0.184 (0.092) |  |  |
| **(B) *S. mansoni*** |  |  |  |  |  |
| (Intercept) |  |  | -3.228 (0.806) |  |  |
| Region | 2 | Bamako (ref) | 0 | 9.729 | 0.0077 |
|  |  | Koulikoro | 4.402 (1.293) |  |  |
|  |  | Ségou | 0.786 (1.063) |  |  |
| Year | 2 | Baseline(ref) | 0 |  |  |
|  |  | F1 | -1.002 (0.339) |  |  |
|  |  | F2 | -0.780 (0.368) |  |  |
| Baseline *S. haematobium* infection | 2 | Uninfected (ref) | 0 |  |  |
|  |  | Light | 1.085 (0.308) |  |  |
|  |  | Heavy | 1.577 (0.367) |  |  |
| Year*Baseline *S. haematobium* infection | 4 | Uninfected (ref) | 0 | 10.800 | 0.0289 |
|  |  | F1:Light | -0.483 (0.396) |  |  |
|  |  | F2:Light | -1.122 (0.423) |  |  |
|  |  | F1:Heavy | -0.411 (0.459) |  |  |
|  |  | F2:Heavy | -1.539 (0.509) |  |  |
| Age | 1 |  | 5.474 (4.688) |  |  |
| Age^2^ | 1 |  | -9.468 (3.969) | 5.763 | 0.0164 |
| Sex | 1 | Male (ref) | 0 | 5.708 | 0.0169 |
|  |  | Female | -0.361 (0.152) |  |  |

**Table B**: Predictors of full follow-up (baseline, one and two years later) in a cohort of children at sentinel sites in Mali, as a function of baseline infection status and covariates. Parameter estimates (on the logit scale) are from a binomial mixed model. χ^2^ and p values are from likelihood ratio tests comparing models with and without the term in question. Age was mean-centred in analysis. ‘ref’ indicates the reference level of each factor.

| **Variable** | **df** | **Parameter estimate (SE)** | | **χ^2^** | **p** |
| --- | --- | --- | --- | --- | --- |
| (Intercept) |  |  | -0.317 (0.315) |  |  |
| Region | 2 | Bamako (ref) | 0 | 8.229 | 0.0163 |
|  |  | Koulikoro | 0.467 (0.524) |  |  |
|  |  | Ségou | 1.325 (0.422) |  |  |
| Age | 1 |  | -35.547 (2.563) |  |  |
| Age^2^ | 1 |  | -14.310 (2.477) | 255.527 | <0.0001 |
| Sex | 1 | Male (ref) | 0 | 0.066 | 0.7973 |
|  |  | Female | -0.029 (0.114) |  |  |
| *S. mansoni* infection | 3 | Uninfected (ref) | 0 | 0.242 | 0.9705 |
|  |  | Light | 0.010 (0.199) |  |  |
|  |  | Moderate | 0.090 (0.249) |  |  |
|  |  | Heavy | 0.102 (0.259) |  |  |
| *S. haematobium* infection | 2 | Uninfected (ref) | 0 | 0.363 | 0.8340 |
|  |  | Light | 0.081 (0.151) |  |  |
|  |  | Heavy | 0.019 (0.187) |  |  |

**Table C:** The effect of parallel changes in *S. mansoni* infection on the intensity of *S. haematobium* re-infection (eggs/10ml) six months after baseline in Senegal, modelled using negative binomial GLMs. Only individuals with *S. haematobium* at baseline that cleared their infection 6 weeks after PZQ treatment are included in the analysis. χ^2^ and p values are from likelihood ratio tests comparing models with and without the term in question. Age was mean-centred in the analysis. ‘ref’ indicates the reference level of each factor.

| **Variable** | **df** | **Parameter estimate (SE)** | | **z** | **Pr(>\|z\|)** | **χ^2^** | **p** |
| --- | --- | --- | --- | --- | --- | --- | --- |
| Intercept |  |  | 0.089 |  |  |  |  |
| Village | 1 | Nder (ref) | 0 |  |  | 73.144 | <0.0001 |
|  |  | Temeye | 6.179 (1.124) | 5.498 | 0.000 |  |  |
| *S. mansoni* change  (BL-6wk-6mo) | 3 | 0-0-1 (ref) | 0 |  |  | 18.603 | 0.0003 |
|  |  | 1-0-0 | -1.681 (0.778) | -2.159 | 0.031 |  |  |
|  |  | 1-0-1 | -1.472 (0.462) | -3.185 | 0.001 |  |  |
|  |  | 1-1-1 | 1.962 (1.487) | 1.320 | 0.187 |  |  |
| Age | 1 |  | -0.245 (0.103) | -2.370 | 0.018 | 5.427 | 0.0198 |
